# Clinical Observations in Private Practice

**Published:** 1851-03

**Authors:** E. B. Haskins

**Affiliations:** Clarksville, Tenn.


### WESTERN JOURNAL

OF

### MEDICINE AND SURGERY.

MARCH, 1851.

Art. I.—*Clinical Observations in Private Practice.* By Dr. E. B. Haskins, of Clarksville, Tenn.

(continued from page 10.)

Observation III.—*Four Cases of Cerebral Disturbance, connected with Enlargement of the Spleen.*

Case I.—On the 14th of October, 1842, I was summoned to see a young gentleman, about 18 years of age, well grown, and of a good constitution. He had been the subject of intermittent fever during the past summer, and up to near the time of the present attack. The chills had been, from time to time, arrested by quinine, but they would soon make their appearance again.

He had never kept his bed longer than a day or two at a time, and his general health was not thought to be impaired. On arriving, I was informed that the patient had had a fit; that he had fallen, insensible, after a hearty supper; that his feet were placed in warm water, and in five or ten minutes consciousness was restored; no convulsions. I found the young man lying on his back; knees slightly flexed; countenance dull and inexpressive; listless—could with difficulty arouse the mind sufficiently to procure answers to questions; respiration slow and heaving; pulse slow and rather full; complains of no pain. On applying the hand to the abdomen, a tumor was felt occupying the left hypochondrium, extending from the cartilages of the ribs half way to crest of ilium, below, and quite to the *linea alba*, in front. The surface of the tumor was even, and gave a flat sound to percussion. Venesection to twelve ounces, four dry cups over the tumor, and a brisk cathartic, constituted the treatment for the time.

This case fell into other hands, and I do not know the treatment pursued; he recovered, however, after several months' confinement.

Case II.—J. H., male subject, aged about 16 years, of spare frame, but of general good health, was attacked to-day (August 1, 1846,) with a fit, on account of which I was called. The "fit" consisted in falling and remaining insensible for a few minutes, and waking up alarmed. He has had chills during the preceding summer and latter spring months, for which he has taken largely of quinine; has lost flesh, and become pale; appetite good; has continued to exercise freely.

Present symptoms—one hour after seizure: Decubitus dorsal; legs extended; skin pale; countenance inexpressive; tongue clean and moist; pulse slow and natural in volume; respiration slow; dullness of mind, answering questions with great hesitation; great dread of dying.

On examination, the spleen was found much enlarged, firm and yielding a flat sound on percussion; no pain on pressure; bowels confined. Calomel grs. v, followed in five hours by a dose of castor oil; hot fomentations over the entire region of the belly.

August 2d.—Medicine has produced several motions from the bowels; patient feels much relieved; condition of spleen the same. He was now put upon the following mixture*—a tablespoonful twice a day, morning and noon—

℞. Ferri sulph., ℈j,

Pulv. columb.,

Pulv. rhei, } ää ʒj,

Zinzib.,

Extr. cinchonæ, ʒss,

Tinct. aloes socot. f., ℥ij,

Aq. cinnam. f. ℥vij. Misce; together with dry friction over the left hypochondriac region morning and night. This case went on to rapid recovery. The chills disappeared in a fortnight; the spleen gradually reduced; color returned to the skin; and in six weeks health was restored.

Case 3.—J. M., ferryman, about 28 years of age, of fine constitution and stout frame; has been the subject of chills for the last two months, for which he had taken several large doses of quinine, and some purgative medicines; has not ceased to labor, except for a few hours at a time; appetite has been very good; was taken to-day, July 9th, 1848, with great prostration of strength and "strange feelings." Decubitus dorsal; knees flexed; pulse slow and rather feeble; respiration slow and labored—more a succession of deep sighs; countenance vacant; eyes fixed upon some object, with disinclination to remove them; attention difficult to arouse; answers questions slowly and with hesitation; says he would cease to

---

*A modification of the "spleen mixture" so highly recommended by Mr. Twining in vascular engorgement of the spleen.—*See Diseases of the Liver and Spleen, by William Twining.*

breathe if he were to fall asleep, that he has to breathe by effort of will; does not know where he is, but knows his family and physician. On conducting further examination by palpation, the spleen was found much enlarged and tender to firm pressure; tongue white and coated, bowels constipated. About five ounces of blood taken by cups from left hypochondrium; warm fomentations over entire abdomen; half dozen pills of comp. ext. of colocynth.

July 10th. Bowels have been freely moved, and patient feels better. The modified spleen mixture was ordered as in case second, with hot fomentations to belly twice a day. Health was restored in five or six weeks.

Case IV.—September 5th, 1849, I was summoned to see a little boy, abont 4 years old, who has had frequent attacks of intermittent fever during the past summer, which were always arrested by quinine, but would recur again in a week or two; general health but little impaired; appetite has been good, and was playful up to the time of the present seizure. I was told by his parents that he had had a "worm fit;" that soon after a hearty dinner he lay down, and as he fell asleep he was taken with the fit; some distortion of features, spasms of the extremities and muscles of the neck, with stertorous breathing; that he was bathed about the face and neck with spirits of camphor; his feet were rubbed, and in a few minutes the "fit went off." I found him lying in his mother's lap, dozing; face pale; respiration natural, except frequent sighing; pulse rather increased in frequency; temperature natural; condition of bowels not known; when aroused speaks hurriedly, and with agitation, but soon falls again to dozing. Abdomen tumid; spleen distinctly felt enlarged, firm, and pressure excited no manifestations of tenderness. He was placed in a warm bath up to the hips; a dose of castor oil and spirits of turpentine was given; and flannel wrung out of hot brandy was applied to the abdomen.

The bath was directed to be used once a day, and a teaspoonful of the spleen mixture twice a day. I did not see this case again until late in the fall, when it was entirely restored to health.

*Reflections.*—I do not remember to have seen, anywhere, such phenomena as are detailed in this Observation, ascribed to enlargement of the spleen. It is true, Andral (Med. Clinic) has recorded two cases of apoplexy in which the spleen was found enlarged, and another where it was very *small* and *dense;* but in all three of his cases the heart was hypertrophied—a more obvious cause of the cerebral affection. Louis* has "examined many subjects who died suddenly, and in an unexpected manner, without being able to decide upon the cause of death from the state of the organs, I (he) always found the spleen very small." What influence, if any, the spleen may have exercised in the production of the sudden and unexpected seizures in those cases observed by Louis, as well as these here recorded, I will not attempt to define, as every thing on this subject could only be received in the light of hypothesis, whilst the physiological relations of that organ are so imperfectly understood. It may not be out of place, however, to remark that the very opposite conditions of *enlargement* and *diminution* of an organ, are not incompatible with *identity* of pathological influence; for an enlargement of the liver, and a certain species of atrophy of that organ (cirrhosis) may alike cause ascites.

It has for a long time been known that apoplexy and palsy are very prevalent in certain marshy districts.

---

*Louis on Typhoid Fever, translated by H. J. Bowditch, M.D.

May not the spleen, in those cases, have been found in a pathological state? The fact that that organ is more *specifically* affected in protracted intermittents, favors at least an affirmative supposition. If the cases detailed in this paper be regarded as apoplectic, they were certainly comparatively light, and would come under the head of what some writers term *transient* or *fugitive* apoplexy. That the state of the spleen and the cerebral phenomena in these cases, stand related as *cause* and *effect*, derives support from the fact, that treatment was directed alone to that organ, and that no recurrence of seizure took place, but, on the contrary, convalescence ensued.

Have other physicians observed similar cases? If they have, I trust they will report them. I know that the attention of *young and inexperienced* physicians is liable to be more exclusively occupied with the organ that presents the most evident signs of disease, and that the *older* members of the profession, relying upon their sagacity, often diagnose hurriedly and without due examination; whilst *all* who do not preserve notes of their cases, may observe similar ones at too distant periods to be connected in the memory, and each individual case is passed over as simply a coincidence; and thus it is obvious that cases of great interest may be wholly lost to the profession. That physicians of age, experience, and *secure local reputation* are very prone to fall into the habit of hasty and careless examinations, is a truth that cannot be too often presented to the minds of those young men who desire to escape the mortification and odium of blunders in diagnosis, and perhaps fatal errors in treatment.

I will further remark, (and beg pardon in advance for this digression), that in protracted chills — after they have assumed a chronic form—I have never found quinine to restore the health of the patient. I have for several years, in such cases, used the mixture, (as used in the last three cases) with decided success. I prescribe its daily use without regard to the chill, and as the health is restored the chills disappear.

*(To be continued.)*

